# Ethical Issues in Caring for Older Persons in Sub-Saharan Africa: A Scoping Review

**DOI:** 10.21203/rs.3.rs-9190458/v1

**Published:** 2026-04-10

**Authors:** Abdulgafar Lekan Olawumi, Zainab Abdulkadir, Abdullahi Kabir Suleiman, Zainab Abdulazeez Umar, Bukar Alhaji Grema, Godpower Chinedu Michael, Abiso Abubakar Mohammed, Umar Faruk Abdullahi, Mahmud Baba Mahmud, Abdulrauf Segun Ibraheem, Amina Danladi Muhammad, Abba Abba Badamasi, Ibrahim Danjummai Gezawa, Muktar Hassan Aliyu, Muhammad Musa Borodo

**Affiliations:** Department of Family Medicine, Aminu Kano Teaching Hospital; Department of Family Medicine, Aminu Kano Teaching Hospital; Department of Family Medicine, Aminu Kano Teaching Hospital; Department of Family Medicine, Aminu Kano Teaching Hospital; Department of Family Medicine, Aminu Kano Teaching Hospital; Department of Family Medicine, Aminu Kano Teaching Hospital; Department of Family Medicine, University of Maiduguri Teaching Hospital; Department of Internal Medicine, Aminu Kano Teaching Hospital; Department of Family Medicine, Aminu Kano Teaching Hospital; Department of Family Medicine, Federal Teaching Hospital; Department of Family Medicine, Aminu Kano Teaching Hospital; Department of Family Medicine, National Hospital; Department of Internal Medicine, Aminu Kano Teaching Hospital; Vanderbilt Institute for Global Health, Vanderbilt University Medical Center; Department of Internal Medicine, Aminu Kano Teaching Hospital

**Keywords:** Ethics, elder abuse, older persons care, resource allocation, sub-Saharan Africa

## Abstract

**Background:**

Rapid population ageing in sub-Saharan Africa (SSA) is unfolding within fragile health systems, weak social protection, and limited geriatric and palliative services, creating ethical challenges in older adult care. This study critically reviews current literature and policy reports, synthesizes key themes, and proposes evidence-based recommendations for policy, practice, and research.

**Methods:**

A comprehensive literature search examined ethical issues in caring for older adults in SSA across healthcare and social care settings. We searched biomedical, ethics, social science, and gray literature sources using population, ethics, care, and geographic terms. English-language studies from 2000 to 2025 addressing ethical dimensions of older adult care were included.

**Results:**

We identified multiple ethical challenges that affect older adult care in SSA, including justice, equity, autonomy, dignity, vulnerability, abuse, and ageism. Healthcare-related concerns involve unmet needs, resource allocation dilemmas, limited palliative and end-of-life care, and age-biased clinical decisions. Socially, weakening family support systems create moral tensions for caregivers. Policy analyses emphasise rights-based, culturally sensitive, and equitable approaches, highlighting the need for integrated ethical, social, and systemic care strategies.

**Conclusions:**

Addressing ethical issues in older care in SSA requires legally enshrined protections, health-system investments, public campaigns to counter ageism, supportive decision-making frameworks that respect cultural contexts and individual rights and strengthened research and surveillance. A right-based, culturally sensitive approach focused on the voices of older persons is essential.

## INTRODUCTION

Population ageing is a global phenomenon with important implications for health systems, social structures, and ethical practice.^1,2^ In Sub-Saharan Africa (SSA), the proportion of older adults is increasing, albeit at a slower pace than in high-income countries, due to improvements in life expectancy and declining fertility rates.^2,3^ While the absolute number of older persons in SSA remains lower than in Europe or North America, their health and social care needs are growing, often in contexts of fragile healthcare systems, limited geriatric expertise, and constrained resources.^1–4^

Ethical challenges in the care of older adults in SSA emerge as healthcare providers, families, and policymakers navigate issues related to access, quality, and fairness.^4^ These ethical considerations are multifaceted. Older adults often face chronic diseases such as hypertension, diabetes, and dementia, which require long-term patient-centered care.^5^ Yet, health systems in SSA are often oriented toward acute care and maternal-child health, leaving older persons underserved.^1,6^ Social norms, cultural expectations, and family dynamics further complicate the provision of ethical care. For example, autonomy and informed consent, foundational principles in Western bioethics, may conflict with communal decision-making practices prevalent in SSA.^7,8^ The ethical tension arises between respecting individual rights and accommodating cultural norms, requiring clinicians and policymakers to navigate delicate moral terrain.^9^ In addition, despite growing scholarly attention to ageing in SSA, ethical issues remain dispersed across disciplines and inadequately synthesized.^10,11^ Existing reviews have largely focused on epidemiology, service delivery, or social support, with limited critical engagement with ethical dimensions.^10–14^ A scoping review with critical analysis is therefore warranted to systematically map the available evidence, examine how ethical issues are framed, and identify gaps to inform research, policy, and practice.^15,16^

The objectives of this scoping review are to map the range of ethical issues related to the care of older persons in SSA, examine how ethical principles such as autonomy, justice, beneficence, and respect for dignity are applied in different care contexts, and critically analyse the influence of cultural, social, legal, and health-system factors on ethical decision-making. The review will also identify evidence gaps and propose priorities for ethical research, policy, and practice in aging care.

## MATERIALS AND METHODS

### Ethical Frameworks Relevant to Geriatric Care in SSA

Understanding ethical issues in older care requires grounding in bioethical theory. Several frameworks are particularly relevant:

**1. Principlism**: It is based on the four pillars of autonomy, beneficence, non-maleficence, and justice, which provides a practical approach to ethical decision-making in healthcare.^17^

### Autonomy

Respecting older adults’ choices in healthcare decisions, including treatment refusal or advance directives, can conflict with familial authority in SSA contexts.^7^

### Beneficence and non-maleficence

Clinicians must balance interventions that promote well-being while minimizing harm, particularly when resource constraints limit available treatments.^8,9^

### Justice

Ethical allocation of scarce resources, such as medications, hospital beds, or specialized geriatric care, requires transparent and culturally sensitive policies.^9^

1. **Virtue ethics**: Emphasizing character traits and moral dispositions, virtue ethics highlights the clinician’s responsibility to act with compassion, empathy, and integrity.^20^ This approach is particularly useful in SSA, where formal protocols may be limited, and ethical practice relies heavily on professional judgment and relational care.
2. **Care ethics**: Care ethics prioritizes relationships, interdependence, and responsiveness to the needs of vulnerable individuals.^10^ Given the centrality of family and community in older care in SSA, care ethics provides a culturally resonant framework that complements principlism while addressing contextual realities.^10, 11^
3. **Public health ethics**: Ethical frameworks in public health emphasize population-level considerations, resource allocation, and equity.^9, 10^ In SSA, public health ethics is critical when addressing systemic challenges affecting older adults, such as access to vaccinations, chronic disease management, and social protection.^8, 9^

### Registration

The protocol for this scoping review was registered on the Open Science Framework (OSF) and is available at https://doi.org/10.17605/OSF.IO/9AXVW.

### Scope and approach

We employed a systematic and transparent search strategy across multiple databases to identify relevant literature according to the PRISMA-ScR guidelines.^18,19^

### Search strategy

We performed a comprehensive literature search to identify relevant studies addressing ethical issues in caring for older persons in SSA. The review focused on older adults as the population of interest, ethical and moral concerns as the core concept, and healthcare and social care settings within SSA (East, West, Central, and Southern Africa) as the context. The primary question guiding the search was: What ethical issues arise in the care of older persons in SSA, and how are these issues addressed within healthcare and social care systems?

**Figure 1:**
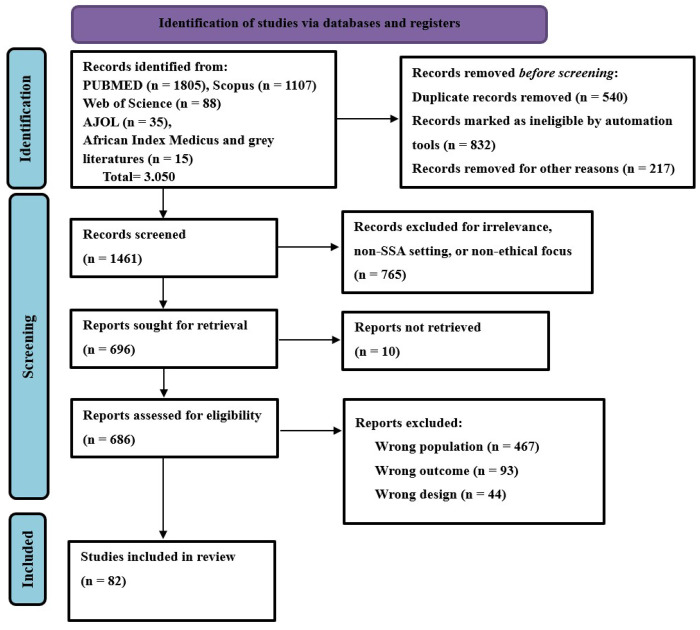
Geographic distribution of studies included in this review across Africa.^1^

Electronic databases searched included PubMed/MEDLINE, Scopus, and Web of Science to capture biomedical and multidisciplinary literature. Given the regional focus, African-specific databases such as African Journals Online (AJOL), and African Index Medicus were also searched. We identified gray literature through targeted searches of reports and policy documents from the World Health Organization (WHO), United Nations agencies, HelpAge International, non-governmental organizations and relevant government sources. The search strategy combined terms related to older persons, ethical issues, care contexts, and geographic location. Terms describing the population included “older persons,” older people,” “older adults,” “elderly,” “ageing,” “aging,” and “geriatrics.” Ethical concepts were captured using keywords such as “ethics,” “bioethics,” “moral issues,” “ethical dilemmas,” “human rights,” “autonomy,” “dignity,” “justice,” “equity,” “ageism,” “elder abuse,” “neglect,” and “exploitation.” Care-related terms included “healthcare,” “health care,” “medical care,” “long-term care,” “social care,” “caregiving,” “nursing care,” and “community care.” Geographic identifiers included “Sub-Saharan Africa,” “Africa South of the Sahara,” and the names of individual countries within the region, such as Nigeria, Ghana, Kenya, South Africa, Ethiopia, Uganda, and Tanzania. Figure 1

In PubMed, both Medical Subject Headings (MeSH) and free-text terms were used to maximize retrieval. Similar keyword-based strategies were adapted for Scopus and Web of Science, using database-specific subject headings and Boolean operators, truncations and wildcards. Searches were limited to articles published in English from the year 2000 to 2025 to reflect contemporary ethical debates and healthcare contexts. Eligible studies included empirical qualitative and quantitative research, systematic and narrative reviews, ethical analyses, and policy papers that explicitly addressed ethical issues in the care of older adults in SSA. Studies were excluded if they focused exclusively on regions outside SSA, did not address ethical or moral dimensions of care, did not focus on older persons, or consisted solely of editorials without substantive ethical analysis.

### Study selection and data extraction

Two reviewers independently screened the titles and abstracts of all identified studies to determine their relevance to the review topic. Studies considered potentially eligible were then retrieved for full-text assessment against the predefined inclusion and exclusion criteria. Any disagreements between the reviewers at any stage of the screening process were resolved through discussion and consensus; where necessary, a third reviewer was consulted to ensure objectivity and consistency in study selection. Data were extracted using a standardized data extraction form to ensure uniformity and completeness. The extracted information included details on authorship, year of publication, and study period, as well as the country and sub-region within Sub-Saharan Africa where the study was conducted. Additional variables included study design (e.g., qualitative, quantitative, or mixed methods), study setting (urban, rural, or mixed), and population group. Given the focus of this review, particular attention was paid to identifying and documenting key ethical issues related to the care of older persons. These included, but were not limited to, concerns around autonomy, informed consent, dignity, confidentiality, access to care, equity, cultural considerations, and end-of-life decision-making. Where reported, information on ethical frameworks, guidelines, or principles applied in the studies was also extracted. In cases where important information was missing, unclear, or insufficiently reported, corresponding authors were contacted for clarification or additional data.

## RESULT

**Figure 2:**
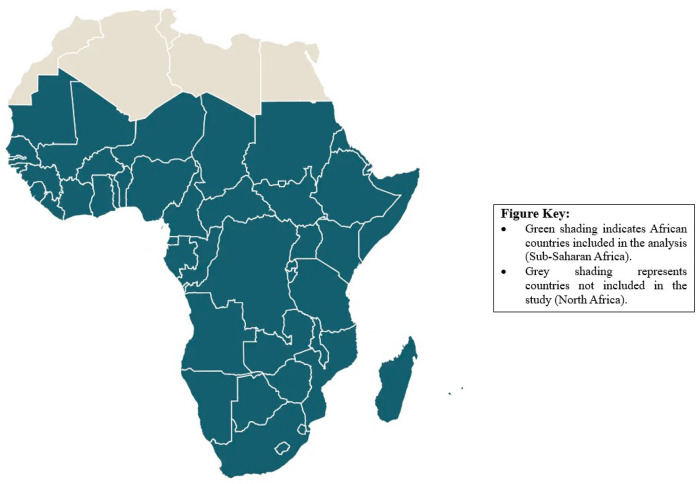
The PRISMA-ScR flow diagram illustrates the study selection process in the scoping review, showing how studies were identified, screened, and assessed for inclusion or exclusion.

As shown in Fig. 2, a total of 82 studies met the inclusion criteria for this scoping review, published between 2001 and 2025. The body of evidence comprised predominantly reviews, qualitative studies, cross-sectional studies, and policy reports focusing on older adults across SSA. Collectively, the findings reveal a wide range of ethical and moral concerns in the care of older adults, with recurrent themes of justice, equity, autonomy through consent and decision making, dignity, vulnerability, ageism, intergenerational responsibility, and culturally grounded ethical frameworks shaping care experiences.

Within healthcare contexts, we found that service delivery for older adults in Nigeria and across SSA face multiple ethical challenges, particularly in decision-making, consent, palliative and end-of-life care (Table 1). In addition, culturally mediated decision making, limited palliative infrastructure, and inadequate professional training frequently undermine ethically-sound care. Qualitative studies from Nigeria by Doobay-Persaud et al. (2023) and Udeh et al. (2025), and a systematic review by Gysels et al. (2011), showed that communication barriers, family-dominated decisions, and poor symptom control often compromise autonomy and dignity at the end of life.^21–23^ Powell et al. (2014) further highlighted the absence of comprehensive palliative care policies, reinforcing systemic inequities in access to compassionate care.^24^ Hospital- and clinic-based studies reveal broader ethical vulnerabilities. Olawumi et al. (2021) documented high morbidity and poor nutritional status among hospitalized older adults in northern Nigeria, raising concerns about neglect and distributive justice.^5^ Odusanya et al. (2018) and Olanrewaju et al. (2020) identified ageism, financial barriers, and fragmented services as persistent constraints to equitable access.^25,26^ Knight et al. (2018) further demonstrated how older adults with HIV and non-communicable diseases (NCDs) navigate poorly coordinated services, highlighting failures in continuity and equity.^27^ Ethical challenges in autonomy and informed decision-making are evident in consent, disclosure, and treatment prioritization. Ede et al. and Mussie et al. (2024) showed that cultural norms privileging family authority and clinician discretion limit individual autonomy, reflecting relational ethics, but risking marginalisation.^28,29^ A report from Ethiopia by Tegegn et al. 2018 found that older patients value involvement in deprescribing, suggestive of tensions between clinical judgement, autonomy, and resource constraints.^30^ In Uganda, Alupo et al. (2025) highlighted systemic and cultural barriers to anticipatory care based on low uptake of advance directives.^31^ Access and stigma also remained critical concerns: Jolley et al. (2025) showed older adults with disabilities in Uganda navigate structural barriers to eye care,^32^ while Wandera et al. (2025) identified stigma as an ethical barrier in the HIV care cascade.^33^ Together, these findings demonstrate that ethical challenges in healthcare delivery for older adults in SSA extend beyond clinical encounters, and reflect deeply embedded structural, cultural, and policy-level constraints.

In social care contexts across SSA, as shown in Table 2, ethical challenges for older adults arise amid changing family structures, migration, poverty, and weakening informal support systems.^14^ Evidence from Ghana, Malawi, and other SSA settings indicate that family-based care, traditionally central to elder support, is increasingly strained Narrative reviews from Ghana by Agyemang (2025) and Dovie (2019), and studies in Democratic Republic of Congo, by Adei et al. (2025), and Bikouta et al. (2015), document strong moral expectations on families and informal caregivers alongside economic hardship, urbanisation, and inter-generational change, resulting in neglect, reduced autonomy, and lower quality of care.^7,34–36^ Hospital-based and community studies in Malawi^37^, and South Africa ^38,39^, highlight risks of malnutrition, abuse, and caregiver burden, reflecting both household- and system-level vulnerabilities.^37–39^ Regional and cross-national analyses, including works by Okolie et al. (2025), Aboderin (2025), HelpAge International (2011), Adamek et al. (2022), and Gedfew et al. (2024), identify gaps in social protection, prevalence of elder abuse, and limited institutional safeguards, situating ethical concerns within broader social and policy environments.^4,40–43^

On the other hand, Jecker (2022) applied an inter-generational ethical perspective, framing moral obligations toward older adults within multi-generational and skipped-generation households, despite structural constraints.^44^ Older adults’ lived experiences further illuminate these ethical challenges: qualitative studies from Uganda,^32^ Nigeria,^45^ and Ethiopia,^46^ reported perceptions of vulnerability, loss of autonomy, and culturally incongruent care. Ethics-of-care approaches, emphasizing relational responsibility, dignity, and context-sensitive care, are also described in studies from South Africa,^47^ and Ghana.^48^ Collectively, the published evidence from Nigeria, Uganda, Ethiopia, Ghana, Malawi, and South Africa consistently link elder abuse and neglect to social inequality, caregiver stress, migration, food insecurity, and limited regulatory oversight.^49–67^

Table 3 illustrates how ethical approaches to ageing in SSA are shaped by global human rights norms, public health ethics, and locally grounded moral obligations, while revealing gaps in implementation, training, and system-level support for older adults. Empirical studies from SSA consistently showed that poverty, inequitable access to care, age-related discrimination, and health system fragmentation remain central ethical concerns shaping ageing experiences.^68–70^

### Global and regional policy frameworks

Global and regional policy instruments, including the WHO Global Strategy on Ageing and Health (2017), the WHO AFRO Framework for Healthy Ageing (2022), and the United Nations World Population Ageing Report (2023), promote rights-based, equitable, and context-sensitive approaches to ageing.^2,71,72^ These frameworks prioritize dignity, autonomy, and equity while advocating integration of health and social care systems. However, evidence from Ghana, Senegal, Nigeria, and Eswatini revealed implementation gaps, with older adults reporting unmet basic needs, financial hardship, and unequal utilisation of healthcare services, despite policy commitments to universal health coverage.^69,70,73–75^

### Ethics of research, policy, and health systems

Expert reports and reviews by the National Research Council (NRC, 2006),^76^ Prince et al. (2015),^77^ Aboderin (2010; 2025),^3,4^ Brock and Wikler (2006),^9^ and Childress et al. (2002),^10^ emphasized justice, distributive fairness, and ethical resource allocation in ageing societies. These ethical concerns are reflected in empirical studies showing socioeconomic inequalities, frailty-related vulnerability, and inequitable service provision among older adults in Ghana, Rwanda, Kenya, and Nigeria.^69,78–81^ Studies further documented structural barriers such as fragmented primary care, inadequate workforce training, and low preventive service uptake, including vaccination among caregivers and healthcare workers.^75,82^

### Human rights, bioethics, and contextual moral frameworks

Comparative rights analyses by Doron et al. (2019) introduce the International Older Persons’ Human Rights Index as a tool for ethical monitoring of elder care systems.^83^ The role of inter-generational obligations, family responsibility, and moral duties toward older adults are highlighted by conceptual and case-based work that advance culturally grounded African bioethics.^13,84^ These perspectives align with findings on ageism, workplace discrimination, and the moral consequences of poverty and multimorbidity in later life.^68,78^ Foundational biomedical ethics texts by Beauchamp et al. (2019) and Brock and Wikler (2006) also stress the core principles of autonomy, beneficence, non-maleficence, and justice, that underpin ethical decision-making in geriatric practice across SSA.^9,17^

## DISCUSSION

This scoping review demonstrates that ethical challenges in the care of older adults in SSA are complex, interrelated, and deeply embedded in clinical, social, cultural, and structural contexts. Across the literature, five recurring ethical domains emerge: informed consent and decision-making capacity; end-of-life and palliative care; resource allocation and justice; elder abuse, neglect, and vulnerability; and cultural considerations under ethical pluralism. Together, these findings reveal systemic gaps that compromise autonomy, dignity, and equity for ageing populations in the region.

### Informed Consent and Decision-making Capacity

Informed consent and decision-making capacity constitute persistent ethical challenges in the care of older adults in SSA, shaped by intersecting clinical, sociocultural, and structural constraints. While autonomy-based bioethical frameworks remain foundational, their application is frequently constrained in practice due to cognitive impairment, frailty, low literacy, and entrenched communal decision-making norms.^9,13,17,51, 81,94^ Evidence from Nigeria and Ghana suggest that clinicians often struggle to obtain informed consent, especially in acute care, surgical, and end-of-life settings.^21 28,95^ In these contexts, consent processes are often abbreviated, poorly documented, or delegated to family members without formal capacity assessments. Such practices, while driven by time pressure and cultural expectations, risk undermining the autonomy of older adults and exposing them to paternalistic decision-making.^25,89,95^

Qualitative studies further show that decision-making in SSA is rarely individualistic. Family hierarchies and inter-generational moral obligations strongly influence healthcare choices, particularly for older adults with cognitive decline or functional dependence.^7,29,34,44^ Jecker’s inter-generational ethics framework argues that surrogate decision-making in African contexts should not be viewed solely as ethical failure, but as relational autonomy embedded within social responsibility.^44^ However, without safeguards, this model may also enable coercion, concealment of diagnoses, or exclusion of older persons from meaningful participation.^49,51,96–97^ Low health literacy and limited geriatric training among healthcare workers further compromise consent validity.^49^ Studies from Ethiopia and Nigeria reveal clinicians’ reluctance to disclose full prognostic information to older patients, citing fears of distress or perceived inability to understand complex information.^21,28, 51,89,95^ While well-intentioned, such non-disclosure conflicts with ethical and rights-based standards articulated by the WHO and the Nuffield Council on Bioethics.^71,91^ Overall, the literature suggests that ethically robust consent in SSA requires culturally responsive models that integrate capacity assessment, supported decision-making, and family engagement while prioritizing the dignity and expressed preferences of older adults. Strengthening geriatric ethics training, developing context-sensitive consent tools, and aligning clinical practice with rights-based frameworks are critical to addressing these challenges.

### End-of-Life Care and Palliative Ethics

End-of-life care in SSA is characterized by substantial ethical challenges rooted in weak health system capacity, including limited availability of palliative care services, poor access to essential analgesics, and shortages of trained healthcare professionals.^22,24^ These structural deficiencies result in inadequate symptom control and delayed initiation of palliative care, and compromise the ethical obligations of beneficence and non-maleficence, especially for frail and functionally dependent older adults.^21^ Ethical dilemmas often arise when clinicians must decide between continuing life-prolonging interventions and transitioning to comfort-oriented care in the absence of clear prognostic guidance or institutional protocols.^99,100^ Evidence from Nigeria and other SSA settings demonstrates frequent discordance between clinicians’ clinical assessments and families’ expectations, with families often favoring maximal intervention due to culturally-embedded beliefs surrounding hope, moral duty, and the social meaning of death.^24,101^ Such tensions may contribute to over-treatment, delayed palliation, and moral distress among healthcare providers operating in resource-constrained environments.^101^

Cultural norms that prioritize collective decision-making and filial responsibility further complicate end-of-life ethics, sometimes marginalizing older adults’ preferences, comfort, and experiential dignity.^13,36^ The ethical complexity of end-of-life care is exacerbated by the limited uptake of advance care planning and the absence of clear legal and policy frameworks guiding surrogate decision-making in many SSA countries.^86,89^ In this context, clinicians are often required to navigate ethically ambiguous decisions with little institutional or legal support. Guidance from the WHO emphasizes the integration of palliative care into primary healthcare systems and advocates for rights-based approaches that prioritize dignity, symptom relief, and culturally-responsive family engagement.^71,72^ Collectively, the literature highlights the need for ethically grounded, context-sensitive end-of-life care models that reconcile clinical judgment, cultural values, and the fundamental right of older adults in SSA to a dignified death.

### Resource Allocation and Justice

Resource limitations are a defining feature of many health systems in SSA, with ethical implications for justice and equitable healthcare delivery for older adults. Evidence from Nigeria and across SSA suggest that older persons experience persistent barriers to accessing healthcare services, essential medicines, and specialized geriatric care, reflecting structural inequities in resource distribution.^81,85^ These constraints disproportionately affect older adults with multimorbidity, frailty, and functional limitations, worsening vulnerability within already resource-limited systems.^5^ Such inequities raise concerns regarding distributive justice, social responsibility, and the moral obligations of health systems toward vulnerable populations.^9,10^

Ethical frameworks emphasize fair, transparent, and needs-based allocation of scarce resources; however, studies consistently identify age-based discrimination (“ageism”) as a significant determinant of healthcare access for older adults in SSA.^26^ Ageism manifests through overt neglect, financial exclusion, and subtle marginalization in clinical prioritization and referral pathways.^25,26,87^ Such practices contravene the principles of justice and non-maleficence, as older adults may be denied interventions that preserve function, alleviate suffering, or improve quality of life.

Ethical tensions related to resource allocation are particularly evident in palliative and end-of-life care. Limited infrastructure, shortages of trained personnel, and competing service priorities frequently compel clinicians to navigate difficult trade-offs between life-prolonging interventions and comfort-focused care, often in the absence of clear institutional guidance.^24,100^ These challenges are exacerbated by the high burden of chronic disease and disability among older adults in SSA, which increases demand for long-term and resource-intensive care.^5^

The literature further highlights that ethical approaches to resource allocation in SSA must be contextually grounded, accounting for sociocultural norms, health system constraints, and population-specific vulnerabilities.^90,69^ Geriatric ethics in African settings requires balancing individual clinical need with collective welfare, while avoiding structural neglect of older persons. Policy-oriented analyses emphasize the need to integrate geriatric-focused strategies into broader health system reforms, including investment in human resources, training in ethical decision-making, and mechanisms to address entrenched inequities in access and quality of care.^9,10,93^

In summary, resource allocation within SSA health systems represents a critical ethical challenge in which principles of justice and equity are frequently compromised. Addressing these challenges requires the deliberate integration of ethical frameworks into policy and practice, prioritization of older adults as a vulnerable population, and sustained investment in geriatric and palliative care infrastructure.

### Elder Abuse, Neglect, and Vulnerability

Elder abuse and neglect in SSA represent significant ethical challenges that intersect with issues of justice, human rights, and societal responsibility. Older adults in SSA are disproportionately vulnerable due to social, economic, and familial pressures compounded by weak social protection systems.^3,41,85,88,95^ The ethical responsibility to protect older adults extends beyond formal healthcare systems to informal care networks, which are often overstretched or under-resourced.^12,44,85,95^ Chronic illnesses, functional disability, and cognitive impairment significantly exacerbate vulnerability, necessitating targeted safeguarding strategies.^5,44,102^ Ethical frameworks emphasize the duty of care owed to older adults, highlighting the moral imperative to prevent harm and promote dignity, particularly in settings where ageism and societal neglect are prevalent.^26,96^ Furthermore, the pervasive nature of elder abuse, ranging from physical and emotional maltreatment to financial exploitation, reflects broader structural inequities, including poverty, limited access to healthcare, and social marginalization.^3,41,85^ These conditions create ethical dilemmas around allocation of resources, prioritization of vulnerable groups, and societal obligations to uphold the rights of older adults.

The literature highlights the importance of a multi-level ethical response: integrating formal health interventions, legal protections, and community-based support systems to reduce elder vulnerability. In SSA, this involves balancing resource constraints with the moral imperative to protect and promote the dignity and wellbeing of older adults, reflecting a justice-oriented approach to ageing care.

### Cultural Considerations and Ethical Pluralism

Sub-Saharan Africa encompasses a rich tapestry of cultural norms, family structures, and belief systems, all of which profoundly influence ethical perspectives and practices in the care of older adults.^3,88,85^ Across all domains, cultural norms profoundly shape ethical practice in SSA. Care for older adults is embedded within family and communal structures, where collective responsibility often supersedes individual autonomy. Ethical pluralism: recognising the legitimacy of multiple moral frameworks, emerges as essential for reconciling biomedical ethics with local traditions.^4,14,44^ Rather than viewing communal decision-making as inherently unethical, the evidence supports approaches that integrate relational autonomy with safeguards against marginalisation.^21–24,96^ Culturally competent care requires policies and practices that support families while ensuring that older adults’ rights, dignity, and wellbeing remain central.^102,103^ Community engagement, intergenerational dialogue, and locally adapted interventions are consistently identified as critical to ethically coherent ageing care in SSA.

### Policy Implications

Addressing ethical challenges in the care of older adults in SSA requires strengthened policy frameworks that integrate ethical principles with public health priorities:

1. Ageing should be explicitly recognized as a major demographic and social transition with distinct healthcare and ethical implications.^3,14^
2. Ethical prioritisation within policy should include the development of age-sensitive national health strategies that incorporate guidance on autonomy, informed consent, end-of-life care, and protection from abuse, while ensuring that healthcare resource allocation does not discriminate against older persons.^34,65^
3. Policymakers should support the integration of geriatric and ethics training into medical and nursing curricula to enhance providers’ capacity to assess decision-making ability, manage ethical dilemmas, and communicate effectively with older adults and their families.^26^
4. Strengthening legal protections for older persons is critical, including enforceable rights related to informed consent, abuse prevention, and access to healthcare. South Africa and Ghana have introduced older-persons legislation, but inconsistent implementation limits their impact, highlighting the need for stronger regulatory and enforcement mechanisms.^6–8^
5. Community engagement and public awareness initiatives can reduce stigma surrounding ageing, cognitive decline, and end-of-life care, while strengthening caregiver support and improving older adults’ access to health and social services through existing community networks.^23,48^

### Research Implications

Ethical ageing in SSA is constrained by limited empirical evidence on older populations, cultural influences on consent, and health system capacity.^54,58,81,97^ Priority research areas include epidemiological studies to guide ethical resource allocation and service planning in ageing populations, and studies of multimorbidity, cognitive impairment, and functional decline.^52,58,102^ Research is also needed to adapt and validate decision-making capacity assessment tools appropriate to SSA contexts, accounting for language, literacy, and cultural norms.^7,25,88^ Comparative studies on ethical frameworks for resource allocation can inform transparent and culturally acceptable prioritization approaches in constrained systems.^9^ Further work on community-based palliative care models and elder abuse prevention strategies is essential to strengthen ethical standards and protective mechanisms.^41,53–55^

### Clinical Practice Implications

At the clinical level, ethical care requires patient-centered, culturally responsive approaches. Clinicians should adopt decision-making models that respect individual autonomy while acknowledging communal decision-making traditions.^17^ Transparent triage processes, early integration of palliative care, and culturally competent communication with families and communities are critical to ensuring ethical, dignified care for older adults in resource-limited settings.^9,22^

## LIMITATION

In this scoping review, we acknowledge some limitations. Restriction to only English-language studies is a threat to language bias. Our findings should be interpreted as descriptive rather than conclusive since the main aim of scoping is to map the current state of knowledge rather than to determine effect sizes or study quality. In spite of these limitations, this review provides a foundational overview of the field ethics in the care of older adults in SSA and highlights important knowledge gaps that warrant future research.

## CONCLUSION

In this scoping review, we found that multifaceted ethical challenges shape the care of older adults in SSA. Key concerns include informed consent, end-of-life care, equitable resource allocation, protection from abuse, and the need for culturally grounded ethical pluralism. Addressing these issues requires integrated approaches that align policy frameworks, professional ethics, family engagement, and cultural competence across healthcare and social care settings. We also found that ethical ageing in SSA depends on bridging clinical ethics, public health ethics, and social justice perspectives. Strengthening ethical care will therefore require rights-based policies, culturally responsive models of care, and shared accountability among families, communities, health systems, and the state to protect the dignity, rights, and well-being of older adults.

## Figures and Tables

**Table 1:** Ethical Issues in Care, Decision-Making, Consent, and End-of-Life Care

Author(s) (Year)	Region	Study Type	Population / Scope	Ethical / Moral Focus	Key Contributions to Ageing Ethics
Doobay-Persaud et al. (2023).^21^	Nigeria	Qualitative study	Health professionals, patients, older adults	Palliative care ethics, dignity, communication	Explores ethical challenges in symptom control, interprofessional communication, and delivery of culturally sensitive palliative care
Powell et al. (2014).^24^	Africa	Commentary	Patients, health systems	Policy ethics, justice, access to palliative care	Highlights ethical and policy challenges in scaling palliative care services in resource-limited settings
Gysels et al. (2011).^22^	SSA	Systematic review (qualitative)	Patients, families, healthcare providers	End-of-life care, dignity, cultural ethics	Synthesizes evidence on ethical issues in end-of-life care, including communication and cultural perspectives
Udeh et al. (2025).^23^	Nigeria	Qualitative study	Patients, healthcare providers	End-of-life decision-making, autonomy, dignity	Examines ethical dilemmas in end-of-life care including consent, truth-telling, and shared decision-making
Tegegn et al. (2018).^30^	Ethiopia	Cross-sectional study	Older patients	Autonomy, deprescribing ethics	Explores older patients’ perceptions of medication withdrawal and ethical considerations in clinical decision-making
Odusanya et al. (2018).^25^	Nigeria	Narrative review	Older adults	Health system ethics, dignity, access to care	Identifies systemic and ethical challenges affecting care quality and equity among older persons
Olanrewaju et al. (2020).^26^	Africa	Narrative review	Older adults	Ageism, dignity, equity	Examines how age-based discrimination affects access to healthcare and ethical care delivery
Knight et al. (2018).^27^	South Africa	Qualitative study	Older adults with HIV	Equity, access, stigma	Highlights barriers to healthcare access and ethical concerns related to stigma and multimorbidity
Ede et al. (2024).^28^	Nigeria	Qualitative study	Surgical patients (including older adults)	Informed consent, autonomy	Assesses ethical challenges in obtaining valid informed consent in resource-limited surgical settings
Mussie et al. (2024).^29^	Ethiopia	Qualitative study	Older patients, clinicians	Disclosure ethics, autonomy	Explores ethical tensions around truth-telling and information disclosure in elderly care
Jolley et al. (2025).^32^	Uganda	Qualitative study	Older adults with disabilities	Access, equity, justice	Examines ethical issues in access to eye care services among vulnerable older populations
Wandera et al. (2025).^33^	SSA	Systematic review protocol	Older adults with HIV	Stigma, confidentiality, equity	Investigates ethical implications of stigma across the HIV care continuum
Alupo et al. (2025).^31^	Uganda	Mixed-methods study	Older adults	Autonomy, advance directives	Explores awareness and ethical implications of advance care planning and decision-making

**Table 2:** Social Care Ethics: Family Responsibility, Elder Abuse, Neglect, and Vulnerability

Author(s) (Year)	Region	Study Type	Population / Scope	Ethical / Moral Focus	Key Contributions to Aging Ethics
Agyemang (2025).^7^	Ghana	Conceptual	Older adults, families	Family ethics, moral obligations	Examines evolving family roles in elder care and the ethical implications of shifting caregiving responsibilities
Dovie (2019).^34^	Ghana	Qualitative	Older adults, families	Family ethics, caregiving norms	Highlights the role of traditional family systems and ethical obligations in supporting older persons
Adei et al. (2025).^35^	Ghana	Cross-sectional	Informal caregivers of older adults	Caregiver burden, social responsibility	Identifies predictors of caregiving strain and ethical concerns related to caregiver support and wellbeing
Bikouta et al. (2015).^36^	Congo	Qualitative	Young people, older adults	Intergenerational ethics, respect	Explores perceptions of ageing and moral attitudes toward older persons among younger populations
Lukman et al. (2025).^14^	Nigeria	Conceptual	Older adults	Structural inequality, social justice	Discusses how social determinants create ethical challenges in ageing and access to care
Mphwanthe et al. (2025).^37^	Malawi	Cross-sectional	Older adults	Vulnerability, nutrition ethics	Examines malnutrition and food insecurity as ethical concerns affecting older adults’ health
Brear et al. (2025).^38^	South Africa	Mixed-methods	Caregivers of older adults	Care ethics, wellbeing	Explores health and wellbeing of caregivers, highlighting ethical dimensions of caregiving burden
Meel (2017).^39^	South Africa	Cross-sectional	Older women	Violence, justice, human rights	Documents sexual violence against elderly women and highlights ethical issues of protection and justice
HelpAge International (2011).^41^	SSA	Report	Older adults	Rights, vulnerability	Provides evidence on systemic neglect and emphasizes ethical obligations for protection and policy response
Adamek et al. (2022).^42^	SSA	Qualitative	Older adults	Social vulnerability, resilience	Explores both challenges and strengths of older adults, emphasizing ethical need for supportive systems
Okolie et al. (2025).^40^	Nigeria	Qualitative	Older adults	Abuse, caregiving ethics	Distinguishes between caregiving and abuse, highlighting ethical grey areas in elder care
Brear et al. (2024).^47^	South Africa	Qualitative	Older adults, caregivers	Ethics of care, quality of care	Conceptualizes “good care” within informal caregiving networks and ethical responsibilities
Awuviry-Newton et al. (2022).^48^	SSA	Review	Caregivers, older adults	Ethics of care, relational ethics	Uses ethics of care framework to understand caregiving experiences
Kelly et al. (2019).^49^	South Africa	Qualitative	Older adults	Dignity, quality of care	Highlights older persons’ experiences of neglect and perceived lack of respect in healthcare
Gedfew et al. (2024).^43^	SSA	Systematic review & meta-analysis	Older adults	Elder abuse, neglect	Synthesizes prevalence and forms of elder abuse, emphasizing need for ethical protection systems
Mussie et al. (2022).^51^	Eastern Africa	Scoping review	Older adults	Ethical issues in care	Provides comprehensive mapping of ethical challenges in elder care in the region
Aboh et al. (2025).^50^	SSA	Scoping review Protocol	Older adults	Neglect, vulnerability	Proposes systematic assessment of elder neglect and ethical implications
Okigbo et al. (2025).^45^	Nigeria / Diaspora	Qualitative	Caregivers	Care ethics, transnational care	Explores ethical dimensions of caregiving across borders, including emotional and financial strain
Sossou et al. (2015).^52^	Ghana	Qualitative	Older women	Abuse, social justice	Links elder abuse to gender inequality and human rights concerns
Ferreira et al. (2008).^53^	South Africa	Qualitative	Older adults	Abuse, marginalization	Highlights systemic neglect, exploitation, and ethical failures in protecting older persons
Awuviry-Newton et al. (2020).^54^	Ghana	Qualitative	Older adults	Neglect, moral responsibility	Explores perceptions and causes of elder neglect within communities
Wamara (2022).^55^	Uganda	Qualitative	Social workers	Abuse, professional ethics	Examines ethical responses and challenges in addressing elder abuse
Wamara et al. (2021).^57^	Uganda	Qualitative	Older adults	Abuse, dignity	Documents lived experiences of abuse and ethical need for voice and protection
Wolde et al. (2022).^56^	Ethiopia	Cross-sectional	Older adults	Abuse, vulnerability	Identifies prevalence and determinants of elder abuse
Chane et al. (2015).^58^	Ethiopia	Qualitative	Older adults	Abuse, dignity, suffering	Presents narratives of abuse and neglect, emphasizing ethical urgency
Atim et al. (2023).^59^	Uganda	Cross-sectional	Older adults	Abuse, neglect	Examines factors associated with elder abuse in community settings
Okojie et al. (2022).^60^	Nigeria	Cross-sectional	Older adults	Abuse, inequality	Compares rural–urban patterns of elder abuse
Ojifinni et al. (2023).^61^	Nigeria	Qualitative	Caregivers	Abuse, caregiving ethics	Explores caregiver perspectives and ethical dilemmas in elder abuse
Okakah et al. (2025).^62^	Nigeria	Cross-sectional	Older adults	Abuse, vulnerability	Assesses prevalence and predictors of elder abuse in rural settings
Yussuf et al. (2014).^63^	Nigeria	Cross-sectional	Older adults	Abuse, neglect	Documents patterns of elder abuse in Northern Nigeria
Fajemilehin et al. (2007).^64^	Nigeria	Qualitative	Older adults	Neglect, poverty	Highlights ethical implications of destitution among the elderly
Cadmus et al. (2015).^65^	Nigeria	Qualitative	Older adults	Abuse, dignity	Explores lived experiences and perceptions of abuse
Cadmus et al. (2012).^66^	Nigeria	Cross-sectional	Older women	Abuse, gender ethics	Examines prevalence and gendered dimensions of abuse
Agunbiade (2019).^67^	Nigeria	Qualitative	Older adults	Abuse, prevention ethics	Explores causes of abuse and strategies for prevention

**Table 3:** Moral and System-Level Ethical Frameworks, and for Older Adults in SSA

Author(s) (Year)	Region	Study Type	Population / Scope	Ethical / Moral Focus	Key Contributions to Ageing Ethics
Kola et al. (2019).^68^	Nigeria	Conceptual	Older adults	Poverty, distributive justice	Challenges moral attribution of poverty and highlights ethical implications of economic vulnerability in old age
Dei et al. (2018).^69^	Ghana	Cross-sectional	Older adults	Equity, healthcare access	Examines inequities in healthcare utilisation, emphasizing ethical concerns around fairness
Agyemang-Duah et al. (2019).^70^	Ghana	Qualitative	Older adults	Financial protection, access	Explores ethical challenges in financing healthcare among poor older adults
Mabuza et al. (2010).^73^	Eswatini	Qualitative	Older adults	Basic needs, dignity	Identifies unmet basic needs and ethical implications for wellbeing and dignity
Olawumi et al. (2023).^81^	Nigeria	Cross-sectional	Older adults	Functional health, equity	Examines determinants of functional capacity, highlighting inequities affecting ageing outcomes
WHO (2017).^71^	Global	Policy framework	Older adults	Rights-based care, dignity	Provides global strategy promoting ethical, rights-based approaches to healthy ageing
WHO AFRO (2022).^72^	SSA	Policy framework	Older adults	Equity, integrated care	Outlines regional framework for ethical and equitable ageing care systems
Chatterji et al. (2008).^85^	Global	Review	Older populations	Health equity, system ethics	Discusses burden of disease and ethical implications for policy and care systems
Taverne et al. (2023).^74^	Senegal	Qualitative	Older adults	Access, financial ethics	Explores healthcare utilisation and ethical issues related to cost and access
Ogunyemi et al. (2025).^75^	Nigeria	Qualitative	Older adults	Service provision, dignity	Examines barriers to primary healthcare and ethical implications for quality care
NRC (2006).^76^	Global	Expert report	Older populations	Research ethics, policy	Provides recommendations for ethical research and policy development in ageing
Prince et al. (2015).^77^	Global	Review	Older populations	Policy ethics, health systems	Highlights burden of ageing and ethical need for responsive health systems
Mba (2010).^86^	Ghana	Review	Older adults	Policy, demographic ethics	Identifies research gaps and policy needs for ageing populations
Aboderin (2010).^3^	SSA	Narrative review	Older adults	Equity, policy ethics	Explores policy gaps and ethical challenges in ageing populations
Aboderin (2025).^4^	SSA	Conceptual	Older adults	Justice, health systems	Updates perspectives on ageing and ethical policy priorities
Brock et al. (2006).^9^	Global	Book chapter	Populations	Resource allocation, justice	Discusses ethical issues in allocation of healthcare resources
Childress et al. (2002).^10^	Global	Conceptual	Public health systems	Public health ethics	Defines ethical principles guiding population-level interventions
Bayuo (2017).^87^	Ghana	Qualitative	Older adults	Access, dignity	Explores healthcare utilisation experiences and ethical concerns
Asiamah et al. (2024).^78^	Ghana	Cross-sectional	Older adults	Frailty, equity	Examines functional decline and equity in ageing outcomes
Jecker & Atuire (2022).^13^	Africa	Case-based analysis	Older adults, families	Contextual bioethics	Advocates for culturally grounded African bioethics frameworks
Jecker (2022).^44^	Africa	Conceptual	Families, older adults	Intergenerational ethics	Explores moral obligations toward older adults in African contexts
Kiplagat et al. (2025).^79^	SSA	Qualitative	Providers, older adults with HIV	Integrated care ethics	Examines ethical challenges in integrating HIV and NCD care
Dzando et al. (2025).^88^	SSA	Qualitative	Older adults	Lived experience, care ethics	Synthesizes perceptions of ageing and care experiences
Beauchamp & Childress (2019).^17^	Global	Conceptual	Healthcare practice	Biomedical ethics principles	Establishes principles of autonomy, beneficence, non-maleficence, and justice
Weise (2016).^84^	Global	Review	Clinical practice	Medical ethics	Simplifies ethical principles for clinical decision-making
Agyemang-Duah (2025).^92^	Ghana	Qualitative	Caregivers, older adults	Care ethics, wellbeing	Explores caregiver experiences and ethical responsibilities
Kalache et al. (2002).^93^	Global	Policy/Conceptual	Older populations	Healthy ageing, equity	Emphasizes compression of morbidity and ethical policy priorities
Monsudi et al. (2015).^89^	SSA	Narrative review	Healthcare systems	Medical ethics gaps	Identifies deficiencies in ethical practice and training
Atuire & Bull (2020).^90^	SSA	Commentary	Health systems	Crisis ethics	Highlights ethical challenges intensified during pandemics
Amberbir et al. (2025).^80^	SSA	Mixed-methods protocol	Older adults	Healthy ageing, system ethics	Proposes strategies to address multimorbidity and ageing needs
Nuffield Council on Bioethics (2001).^91^	Global	Ethics report	Vulnerable populations	Research ethics, justice	Provides guidance on ethical conduct in health research
Doron et al. (2019).^83^	Global	Comparative analysis	Older adults	Human rights	Introduces framework for assessing rights of older persons
Sibanda et al. (2023).^82^	South Africa	Cross-sectional	Healthcare workers	Professional ethics	Examines ethical issues in vaccination practices among caregivers

## Data Availability

All data generated or analysed during the study are available and included in this manuscript.
